# Comparison of the Effective Reproduction Number (Rt) Estimation Methods of COVID-19 Using Simulation Data Based on Available Data from Iran, USA, UK, India, and Brazil

**DOI:** 10.34172/jrhs.2022.94

**Published:** 2022-11-19

**Authors:** Ali Karamoozian, Abbas Bahrampour

**Affiliations:** ^1^Department of Biostatistics and Epidemiology, Kerman University of Medical Sciences, Kerman, Iran; ^2^Modeling in Health Research Center, Institute for Futures Studies in Health, Kerman University of Medical Sciences, Kerman, Iran; ^3^Adjunct Professor of Griffith University, Brisbane, QLD, Australia

**Keywords:** COVID-19, Effective reproduction number (Rt), Maximum likelihood estimation, Time-dependent, Simulation

## Abstract

**Background:** Accurate determination of the effective reproduction number (*R*_t_) is a very important strategy in the epidemiology of contagious diseases, including coronavirus disease 2019 (COVID-19). This study compares different methods of estimating the *R*_t_ of susceptible population to identify the most accurate method for estimating *R*_t_.

**Study Design:** A secondary study.

**Methods:** The value of *R*_t_ was estimated using attack rate (AR), exponential growth (EG), maximum likelihood (ML), time-dependent (TD), and sequential Bayesian (SB) methods, for Iran, the United States, the United Kingdom, India, and Brazil from June to October 2021. In order to accurately compare these methods, a simulation study was designed using forty scenarios.

**Results: **The lowest mean square error (MSE) was observed for TD and ML methods, with 15 and 12 cases, respectively. Therefore, considering the estimated values of *R*_t_ based on the TD method, it was found that *R*_t_ values in the United Kingdom (1.33; 95% CI: 1.14-1.52) and the United States (1.25; 95% CI: 1.12-1.38) substantially have been more than those in other countries, such as Iran (1.07; 95% CI: 0.95-1.19), India (0.99; 95% CI: 0.89-1.08), and Brazil (0.98; 95% CI: 0.84-1.14) from June to October 2021.

**Conclusion:** The important result of this study is that TD and ML methods lead to a more accurate estimation of *R*_t_ of population than other methods. Therefore, in order to monitor and determine the epidemic situation and have a more accurate prediction of the incidence rate, as well as control COVID-19 and similar diseases, the use of these two methods is suggested to more accurately estimate *R*_t_.

## Background

 The epidemiology of the contagious disease has changed over time, which refers to improved clinical interventions, as well as emerging threats, such as antimicrobial resistance. These diseases lead to widespread mortality worldwide each year.^1-3^ One of the most dangerous contagious diseases of the coronavirus family is coronavirus disease 2019 (COVID-19), the outbreak of which has occurred since late 2019. The transmission rate of this disease is very high and is spreading rapidly in the world.^4-6^ Estimation of the epidemiological features of contagious diseases, including COVID-19, is critical to assessing its prevalence in terms of transmission, predicting future outbreaks, and the effectiveness of disease control strategies.^7-9^ Determination of effective reproduction number (*R*_t_) is very important in monitoring and determining the epidemic situation of contagious diseases, as it determines the transmission rate of the disease.^10,11^ The average number of secondary people infected by primary people is called the basic reproduction number (R0).^12,13^ To determine the basic reproduction number (R0), it should be noted that the target population is susceptible to disease.^14,15^ Due to the fact that in this period, the population in the mentioned countries is not completely susceptible, instead of estimating R0, we estimated *R*_t_. To this end, several studies have been conducted, and the estimation of this index is based on existing methods. Attack rate (AR), exponential growth (EG), maximum likelihood (ML), time-dependent (TD), and sequential Bayesian (SB) methods available in susceptible-infectious-removed (SIR) and susceptible-exposed-infectious-removed (SEIR) multi-component models are the most important and most practical of these methods.^16,17^ The important point here is that in most studies, randomly or arbitrarily, one or more methods have been used to determine R0 and *R*_t_.^18-23^ The purpose of comparing the methods studied in different countries is to check which method is known as the most accurate method in different data conditions of different countries. Another reason for this comparison is to compare the *R*_t_ values of different countries to check in which countries the epidemic situation occurred between June and October 2021. Moreover, it aims to investigate whether this reduction was more noticeable in countries that had a more comprehensive vaccination program in this period.

 Considering the fact that there is a relatively large difference between the estimated values of *R*_t_ using these methods, as well as the difficulty of choosing the most accurate *R*_t_, one of the challenging issues in these studies is whether the chosen method was the best one, and the estimated value of *R*_t_ was the most accurate. The answer to this question definitely requires a comprehensive and accurate study to use simulated data as well as real data, compare all these methods, and determine the most accurate method. Therefore, in the present study, in addition to a complete comparison of *R*_t_ values of different methods in different countries, simulated data have been used to determine the best and most accurate method using different scenarios. On the other hand, by using this index and choosing the best method, as well as achieving the exact effective reproduction number, it will be possible to detect the epidemic status of this virus.

## Methods

 The present study used five methods, namely AR, EG, ML, TD, and SB to estimate *R*_t_. These methods are available in R0 statistical package, and to implement each of them, the required data were extracted from similar studies conducted over the same period in that country.^24^ To this end, information including the time of onset, peak, and end of the epidemic was needed, which was determined using available data and sensitivity analysis. Moreover, it was necessary to accurately determine the generation time distribution, for which, according to similar studies, gamma distribution with different parameters was used.^25-29^ In addition to this information, AR values based on similar studies were extracted for each of the studied countries and placed in the AR method.^30-33^ Finally, after determining *R*_t_ using default and optimal approaches in different countries, these methods were compared using simulated data based on different scenarios. In the default approach, the same period of time was considered the length of the epidemic for all countries. However, in the optimal approach, using the appropriate commands in the used package, according to the number of daily items and available data, it was possible to consider a separate epidemic length in the analysis for each country.

###  Data

 This study used two data sets. Actual COVID-19 data from Iran, the United States, the United Kingdom, India, and Brazil were collected on a daily basis from the Worldometers site, and other data, as mentioned before, were extracted from similar studies. These data are related to the period from the beginning of June to the end of September 2021, in other words, these data are related to the time when the new Omicron variant had not been yet identified in the world. The reason for selecting these countries was to compare Iran with four countries with the highest outbreak in this four-month period. Other data are simulated data based on different scenarios described in detail below.

###  Statistical analysis

 As mentioned before, the applied statistical models in this study are AR, EG, ML, TD, and SB models, which are available in R0 statistical package. In this study, precise programming in R software was also used to estimate *R*_t_, as well as simulate data, and then compare different models in addition to R0, EpiEstim, and EpiCurve packages.

###  Models

####  Attack rate method (AR)


(1) $$R_{0}=-\left(\frac{\log\left(\frac{1-AR}{S_{0}}\right)}{AR-\left(1-S_{0}\right)}\right)$$

In equation (1), AR is the attack rate, and *S*_0_ presents the initial percentage of the susceptible population.^18,34^

####  Exponential growth method (EG)


(2) $$R=\frac{1}{M\left(-r\right)}$$

In equation (2), *M* is the Moment-generating function of generation time distribution. *r* is also an estimated parameter by Poisson regression.^35^

####  Maximum likelihood method (ML)


(3) $$R=\frac{\sum_{i=1}^{t}N_{t-i}w_{i}}{\mu_{t}}$$

In equation (3), let *N*_0_*, N*_1_*,…, N*_t_ identify incident cases over sequential time, and *w*_i_ is related to the GT distribution. *μ*_t_ is also related to the Poisson distribution parameter obtained by maximizing

$ll\left(R\right)=\sum_{t=1}^{T}\log\left(\frac{e^{-\mu_{t}}\mu_{t}^{N_{t}}}{N_{t}\text{!}}\right)$ .^36,37^

####  Time-dependent method (TD)


(4) $$R=\frac{1}{N_{t}}\sum_{t_{j}=t}R_{j}$$

In equation (4), *R*_j_ is the effective reproduction number for the j^th^ person obtained from

$R_{j}=\sum_{i=1}^{n}\frac{N_{i}w\left(t_{i}-t_{j}\right)}{\sum_{i\neq k}N_{i}w\left(t_{i}-t_{k}\right)}$ .^38^

####  Sequential Bayesian method (SB)


(5) $$P\left(R\left|N_{i}\right.\right)=\frac{L\left(R;N_{i}\right)P\left(R\right)}{P\left(N_{i}\right)}=\frac{P\left(N_{t+1}\left|R,N_{0},...,N_{t}\right.\right)P\left(R\left|N_{0},...,N_{t}\right.\right)}{P\left(N_{i}\right)}$$

In equation (5), *N*_0_*, N*_1_*,…, N*_t + 1_ follows the Poisson distribution. This equation is completely different from the previous one since in order to estimate the effective reproduction number (*R*_t_), classical inference logic is used in equations 1 to 4. In contrast, Bayesian inference is used in equation 5. In the above equation, *P( R │ N*_i_) is the posterior probability distribution, *L*(*R;N*_i_)signifies the likelihood function, and *P(R)* presents the prior probability distribution, which is determined based on the posterior probability distribution of the previous days. Here, the value of R is estimated based on the maximum of the posterior probability distribution function.^21,39^

###  Simulation study

 In order to compare the studied methods, as well as to achieve the most accurate method in estimating the effective reproduction number (*R*_t_), we designed a simulation study based on different scenarios. In this study, in order to increase the similarity of the simulated data to the real data, the data of five countries (Iran, USA, UK, India, and Brazil) were used to design different scenarios. These scenarios were designed considering the generation time distribution (GT), as well as the distribution of new cases, according to the dispersion status.

 The gamma distribution (4.55, 3.30) as the GT distribution and the epidemic interval of 60 days with a peak at day 40 were used in scenarios 1-8; the gamma distribution (4.70, 2.90) and the epidemic interval of 54 days with a peak at day 36 were used in scenarios 9-16; the gamma distribution (5.00, 2.24) and the epidemic interval of 20 days with a peak at day 10 were used in scenarios 17-24; the gamma distribution (6.00, 3.80) and the epidemic interval of 40 days with a peak at day 21 were used in scenarios 25-32; and in scenarios 33-40, the gamma distribution (3.97, 3.29) and the epidemic interval of 41 days with a peak at day 30 were utilized. In each of these 40 scenarios, the negative binomial distribution or Poisson distribution was employed as the distribution of new items, respectively. Therefore, the epidemic started with one case at time t = 0, and then, for each case, the secondary cases were generated based on these two distributions in different scenarios. Moreover, for each of these scenarios, four values were used for *R*_t_ (1, 1.5, 2, and 3). In total, 10 000 epidemics with more than 50 cases were simulated for each scenario. Finally, epidemic data were collected daily, as well as cumulatively for 7 days, and the methods were compared by calculating relative bias and MSE; furthermore, a method was selected as the superior one that in addition to its low relative bias value in estimating *R*_t_, it had the lowest MSE.

## Results

###  Application

 According to the purpose of the study, first of all, *R*_t_ values were estimated for all five countries using different methods. To this end, default and optimal approaches were used. As expected, in the optimal approach, there is no significant difference in the estimated values of *R*_t_ among the different methods. In this period, it can be said that the highest *R*_t_ belongs to the UK and USA. The estimated *R*_t_ values based on the TD method showed that the *R*_t_ values for the UK (1.33; 95% CI: 1.14-1.52) and USA (1.25; 95% CI: 1.12-1.38) are substantially higher than those in other countries, such as Iran (1.07; 95% CI: 0.95-1.19), India (0.99; 95% CI: 0.89-1.08), and Brazil (0.98; 95% CI: 0.84-1.14) from June to October 2021. On the other hand, according to the estimated *R*_t_ values greater than 1, it can be said that during this period, an epidemic situation has been occurred in these two countries, as well as Iran (Table 1). However, there are still differences among the different methods of estimating *R*_t_, and the reader may be hesitant to choose the most accurate indicator in the *R*_t_ estimation. To answer this question and choose the most accurate method, we used the simulation study, the results of which are presented in Table 2.

**Table 1:** Estimation in effective reproduction number *R*_t_ (95% CI) by five different methods in five countries

**Method**	**Time-dependent**	**Sequential Bayesian**	**Maximum likelihood**	**Exponential growth**	**Attack rate**
Iran					
Default	1.04 (0.90, 1.19)	0.99 (0.87, 1.14)	1.02 (1.00, 1.04)	1.03 (1.03, 1.03)	1.13 (1.12, 1.13)
Optimal	1.07 (0.95, 1.19)	1.08 (0.93, 1.24)	1.09 (1.07, 1.11)	1.07 (1.07, 1.08)	1.13 (1.12, 1.13)
USA					
Default	1.12 (1.02, 1.23)	1.05 (0.94, 1.16)	1.06 (1.05, 1.07)	1.10 (1.10, 1.02)	1.09 (1.08, 1.09)
Optimal	1.25 (1.12, 1.38)	1.27 (1.09, 1.46)	1.28 (1.26, 1.30)	1.34 (1.33, 1.35)	1.09 (1.08, 1.09)
UK					
Default	1.13 (0.99, 1.27)	1.16 (1.01, 1.31)	1.05 (1.04, 1.07)	1.04 (1.04, 1.05)	1.09 (1.09, 1.10)
Optimal	1.33 (1.14, 1.52)	1.16 (0.79, 1.54)	1.57 (1.49, 1.65)	1.44 (1.40, 1.50)	1.09 (1.09, 1.10)
India					
Default	0.96 (0.86, 1.05)	0.80 (0.72, 0.88)	1.01 (0.99, 1.02)	0.94 (0.93, 0.94)	1.14 (1.14, 1.15)
Optimal	0.99 (0.89, 1.08)	0.99 (0.84, 1.16)	1.13 (1.10, 1.16)	0.99 (0.98, 1.00)	1.14 (1.14, 1.15)
Brazil					
Default	1.00 (0.89, 1.11)	0.89 (0.83, 0.94)	0.99 (0.98, 1.01)	0.94 (0.93, 0.94)	1.16 (1.15, 1.16)
Optimal	0.98 (0.84, 1.14)	0.80 (0.67, 0.95)	1.06 (1.03, 1.09)	0.96 (0.95, 0.97)	1.16 (1.15, 1.16)

**Table 2:** Mean square errorand relative bias of effective reproduction number (*R*_t_) estimation of each method

**Generation time** **distribution**	*R*_t_	**MSE, Relative bias**									
		**Time-dependent**		**Sequential Bayesian**		**Maximum likelihood**		**Exponential growth**		**Attack rate**	
		**MSE**	**Percent**	**MSE**	**Percent**	**MSE**	**Percent**	**MSE**	**Percent**	**MSE**	**Percent**
G (4.55, 3.30)											
Poisson	1	0.66	44.52	0.21	26.36	0.04	16.67	0.06	19.61	0.02	11.43
NB		0.64	44.19	0.22	25.76	0.08	22.18	0.14	27.06	0.02	11.43
Poisson	1.5	0.07	9.37	0.22	23.31	0.10	23.66	0.11	20.10	0.13	32.74
NB		0.20	21.26	0.14	0.11	0.13	28.98	0.14	27.77	0.14	32.74
Poisson	2	0.09	12.42	0.02	2.53	0.73	74.06	0.65	65.43	0.76	76.99
NB		0.05	0.55	0.29	23.30	0.41	46.09	0.28	34.05	0.76	76.99
Poisson	3	0.67	36.80	1.37	62.16	2.75	123.40	2.34	103.90	3.50	165.50
NB		0.82	41.58	1.70	72.41	2.78	124.70	2.40	106.20	3.50	165.50
G (4.70, 2.90)											
Poisson	1	0.35	36.39	0.90	48.61	0.02	10.55	0.02	8.34	0.08	8.17
NB		0.92	48.66	0.26	27.33	0.11	24.98	0.19	30.17	0.08	8.17
Poisson	1.5	0.13	14.87	0.30	26.29	0.08	18.39	0.04	3.02	0.17	37.61
NB		0.11	12.02	0.28	25.41	0.11	24.48	0.30	47.06	0.17	37.61
Poisson	2	0.06	4.00	0.07	6.45	0.65	66.52	0.13	19.62	0.83	83.49
NB		0.12	9.30	0.28	19.98	0.39	37.74	0.19	25.31	0.83	83.49
Poisson	3	0.32	112.20	1.18	55.12	2.52	112.20	2.06	91.32	3.65	175.20
NB		0.48	27.33	1.54	65.65	2.53	112.30	2.08	92.06	3.65	175.20
G (5.00, 2.24)											
Poisson	1	2.53	60.78	3.65	66.22	0.29	32.43	0.24	25.59	0.01	8.76
NB		2.63	63.37	0.97	41.79	1.84	57.21	2.73	61.88	0.01	8.76
Poisson	1.5	0.09	17.17	0.80	38.65	0.16	11.97	0.28	9.53	0.16	36.74
NB		1.89	45.75	2.18	49.43	0.16	8.54	0.30	4.40	0.16	36.61
Poisson	2	1.59	36.18	1.14	34.53	0.19	0.65	0.36	3.52	0.82	82.31
NB		2.23	37.30	0.52	7.36	0.62	24.90	1.39	51.05	0.82	82.31
Poisson	3	3.64	37.68	0.31	13.68	0.16	4.82	0.68	18.26	3.62	173.50
NB		0.74	13.02	0.08	4.82	1.22	47.71	1.22	39.47	3.62	173.50
G (6.00, 3.80)											
Poisson	1	0.83	47.17	2.15	59.41	0.07	19.16	0.65	44.38	0. 20	12.51
NB		2.26	59.84	0.59	36.02	0.42	38.99	0.64	44.04	0. 20	12.51
Poisson	1.5	0.46	28.88	0.13	(3.35	0.06	4.46	0.11	12.28	0.13	31.12
NB		0.39	26.32	1.07	40.59	0.08	10.46	0.23	0.60	0.13	31.12
Poisson	2	0.25	15.36	0.46	24.75	0.24	24.53	0.32	1.27	0.73	74.82
NB		0.81	28.88	0.35	2.20	0.08	6.89	0.10	3.15	0.73	74.82
Poisson	3	0.11	6.54	0.62	19.66	1.03	49.85	0.33	10.95	3.45	162.20
NB		0.44	21.85	0.11	9.21	2.87	117.40	0.35	3.75	3.45	162.20
G (3.97, 3.29)											
Poisson	1	0.35	36.47	1.06	50.62	0.02	12.13	0.02	8.73	0.03	13.64
NB		0.86	47.89	0.32	29.18	0.01	27.80	0.26	33.33	0.02	13.64
Poisson	1.5	0.12	14.72	0.41	9.38	0.07	22.46	0.10	13.21	0.12	29.42
NB		0.10	11.56	0.37	28.33	0.10	22.45	0.13	22.05	0.12	29.42
Poisson	2	0.06	4.93	0.07	10.27	0.59	60.13	0.49	49.36	0.71	72.56
NB		0.09	7.32	0.27	15.34	0.26	32.63	0.14	19.76	0.71	72.56
Poisson	3	0.42	26.80	1.02	48.59	2.30	101.90	1.79	79.86	3.39	158.80
NB		1.23	56.74	0.57	32.39	3.39	158.20	3.33	154.00	3.39	158.80

Abbreviations: MSE,Mean square error; NB, Negative binomial.

**Figure 1:**
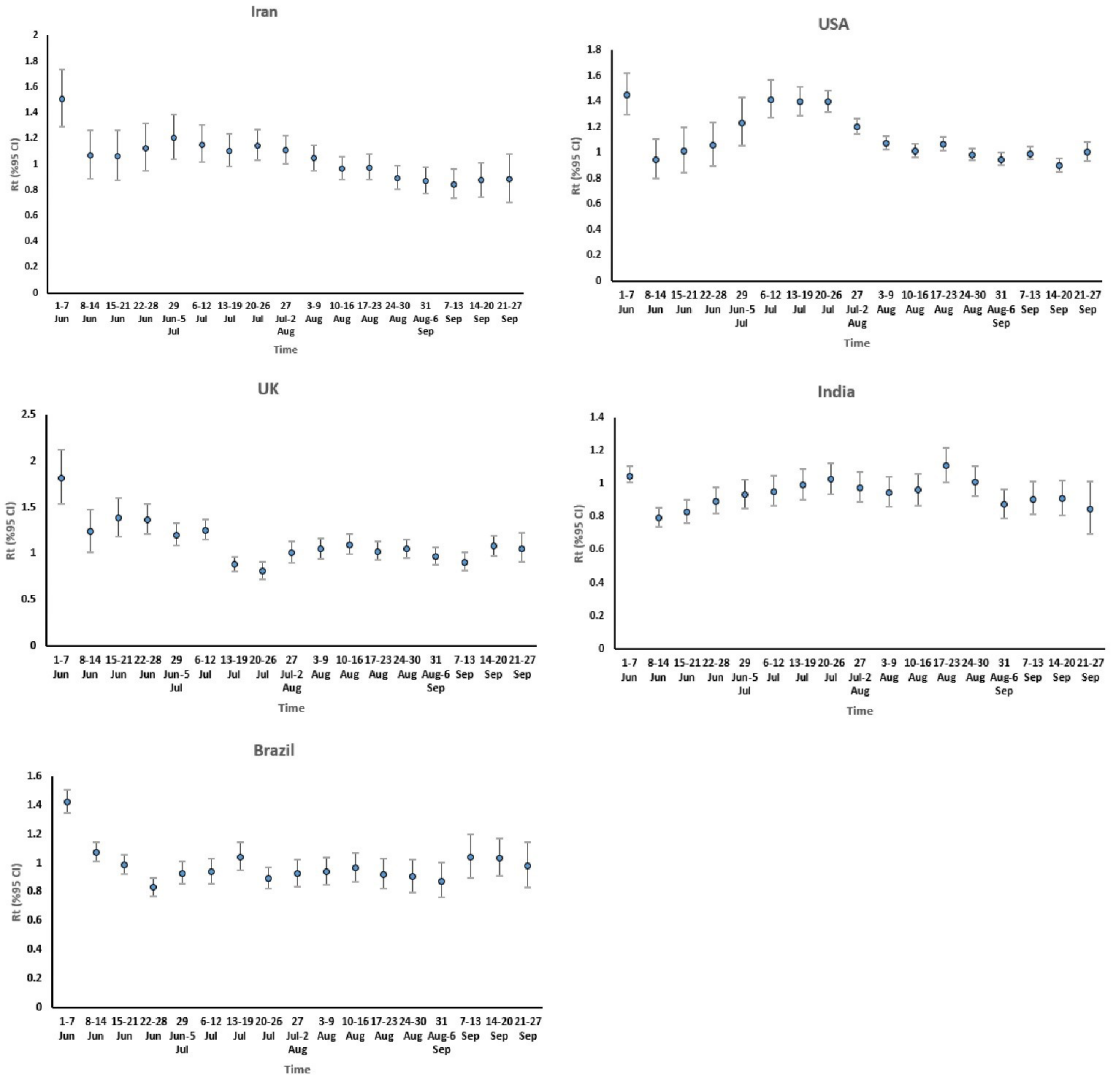
Estimated *R*_t_ with 95% CI in Iran, the USA, the UK, India, and Brazil using a non-parametric model

 In addition to estimating *R*_t_ over a determined period of time (beginning of June to the end of September 2021), these values were also estimated with the corresponding weekly confidence interval, and the results for all five countries are presented in Figure 1. According to the *R*_t_ trend in these graphs, it can be said that in all countries, in general, there is a non-linear trend for this interval. However, according to the results of these graphs, the epidemic interval of each country can be determined approximately.

**Figure 2:**
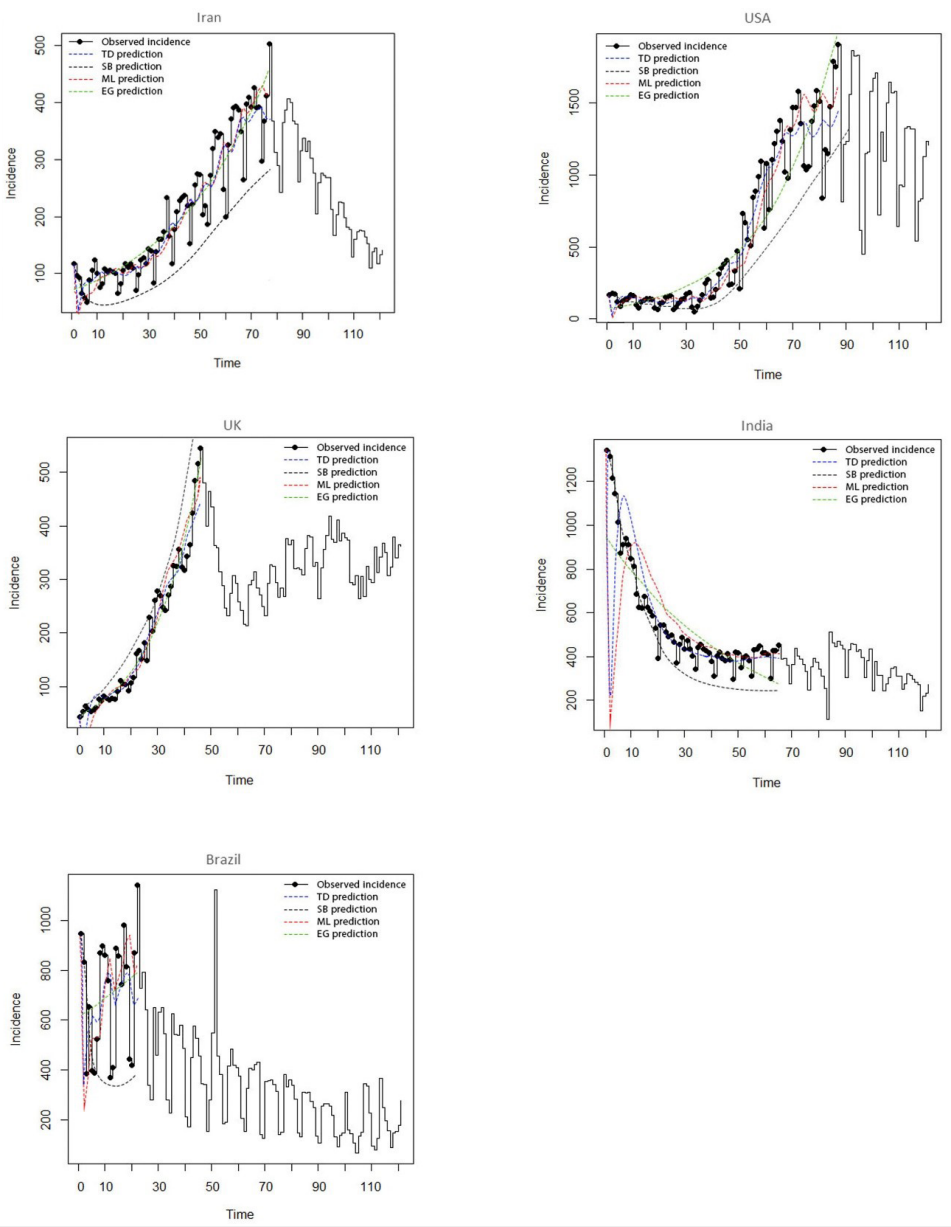
Observed and model predicted incidence for each method in five countries

Figure 2 compares different methods for predicting incidence. As can be seen, the SB method, unlike other methods, underestimates or overestimates the incidence rate for all five countries. On the other hand, it can be said that despite the closeness of the estimation in the three methods, the estimated values in the TD method are much closer to the observed values. However, in order to determine the most accurate method, and whether the TD is really the best method for estimating *R*_t_, a simulation study was designed, the results of which can be seen in the next section.

###  Comparison of methods

 Various scenarios were designed to compare the studied methods. These scenarios are designed according to the desired distributions, as well as different values of *R*_t_. The reason for designing these scenarios is dealing with different conditions contained in the COVID-19 data for a more accurate comparison of the used methods. The results of this simulation study can be seen in Table 2. In this study, the values of MSE and relative bias were determined for each method in different scenarios, and then, the method with the lowest value of MSE was selected as the best method in estimating the *R*_t_ of population. In a total of 40 scenarios, the lowest values of MSE for TD, SB, ML, EG, and AR methods were observed in 15, 6, 12, 1, and 6 cases, respectively. Therefore, considering the results of this study, it can be said that in all scenarios, there was the lowest amount of MSE in the TD method. The next method is the ML, which had the lowest MSE in a greater number of scenarios, compared to other methods.

## Discussion

 Comparing *R*_t_, in different countries, it can be said that this value in the studied interval in Iran, is better than the UK and the USA, while its value is worse than Brazil and India. However, given the value of *R*_t_, it can be said that during this interval, an epidemic situation has occurred in Iran, the UK, and the USA. The interesting point is that the differences among the *R*_t_s in different countries are commensurate with the situation of the COVID-19 epidemic in all these countries. In the period from June to October 2021, the highest weekly value of *R*_t_ is also related to the UK. Perhaps the main reason can be attributed to the reduction of restrictions in this country, compared to other countries in this interval. On the other hand, according to the estimated values of *R*_t _in different countries in this period, it can be claimed that vaccination has a significant effect on reducing the estimated value of *R*_t_, thereby controlling the disease^40^ since during this period, in some countries, one dose, and in some others, two doses of the vaccine was injected.

 A comparison of the studied methods showed that in general, the SB method underestimates and overestimates the incidence rate, while this is not the case with other methods, the prediction of these methods for the incidence of COVID-19 is acceptable. Therefore, it can be expected that these methods, due to their accurate and acceptable prediction, are more appropriate methods for estimating *R*_t _of susceptible populations. However, in order to give an accurate answer to the question of which method actually offers a more accurate estimate of *R*_t_ than others, a simulation study has been used.

 Although there are different methods to estimate *R*_t_, no specific and unique method has been identified that is superior to other methods. However, it can be said that five methods of TD, SB, ML, EG, and AR are used to estimate *R*_t_ more than other methods.^41-46^ Therefore, the aim of the present study was to compare these methods and identify the most accurate method to estimate the *R*_t_ of population. According to the main purpose of the present study, which is to compare the studied methods in estimating the *R*_t_ of population, a simulation study was designed. This study tried to design different scenarios, different aspects, and existing data, to more accurately compare the studied methods.

 In this study, considering MSE values, it was found that the estimation of *R*_t_ in the TD and ML methods are more accurate than that in other methods. The main reason for this superiority can be attributed to the GT distribution, because the appropriate and accurate distribution of GT is particularly important in estimating *R*_t_, and this distribution in the TD is more completely selected and used than in other methods. Other reasons for this superiority include the incidence of new cases, which in this method are considered in an epidemic situation, while this is not the case with other methods. Moreover, TD method requires fewer details of data and parameters than other methods, which is an advantage.^47^ On the other hand, as it is known, the logic of ML method to estimate *R*_t_ is the MLE method, which is a well-known and acceptable method with minimum bias in statistical analysis.^48^ Here, according to the research results, it can be said that the SB method, unlike the AR, which is a suitable method for non-epidemic status, is a relatively acceptable method for epidemic status. In addition, the reason for the poor performance of the EG method to estimate *R*_t_ can be attributed to the low data dispersion, because this method is acceptable when the data are highly dispersed.^34,35^

**Highlights**

- The estimation of *R*_t_ of patients susceptible to COVID-19 using TD and ML methods is more accurate than SB, EG, and AR methods.
- TD and ML methods are more accurate than SB, EG, and AR methods in predicting the incidence rate of COVID-19.
- The epidemic situation occurred in Iran, the UK, and the USA from June to October 2021.

## Conclusion

 According to the research findings and results, it can be concluded that, in order to estimate *R*_t_, the use of the most accurate method is better than using the most common one. According to the present study, there is a relatively large difference between the *R*_t_ estimates in different methods, and this difference highlights the importance of the present study in comparing these methods. Therefore, the general and important result of this study is that TD and ML methods provide a more accurate estimate of *R*_t_ of susceptible population than other methods. Therefore, in order to monitor and determine the epidemic situation, as well as control COVID-19 and similar diseases, the use of these two methods to more accurately estimate *R*_t_ is suggested. Moreover, TD and ML methods are more accurate than SB, EG and AR methods in predicting the incidence rate of COVID-19.

## Conflict of interest

 The authors declare no conflict of interest.

## Ethical approval

 This manuscript with the code IR.KMU.REC.1400.152has been approved by the Ethics Committee ofKerman University of Medical Sciences, Kerman, Iran.

## Funding

 This study received no specific grant from any funding agency in the public, commercial, or not-for-profit Sectors.
